# The Characteristics of Herpes Simplex Virus Type 1 Infection in Rhesus Macaques and the Associated Pathological Features

**DOI:** 10.3390/v9020026

**Published:** 2017-01-30

**Authors:** Shengtao Fan, Hongzhi Cai, Xingli Xu, Min Feng, Lichun Wang, Yun Liao, Ying Zhang, Zhanlong He, Fengmei Yang, Wenhai Yu, Jingjing Wang, Jumin Zhou, Qihan Li

**Affiliations:** 1 Institute of Medical Biology, Chinese Academy of Medical Sciences & Peking Union Medical College, Yunnan Key Laboratory of Vaccine Research and Development on Severe Infectious Diseases, Kunming 650118, China; fst@imbcams.com.cn (S.F.); fst0007@163.com (H.C.); xinglixu@imbcams.com.cn (X.X.); fengmin@imbcams.com.cn (M.F.); wlc@imbcams.com.cn (L.W.); liaoyun@imbcams.com.cn (Y.L.); zhangy@imbcams.com.cn (Y.Z.); wangjingjing@imbcams.com.cn (J.W.); 2 Institute of Medical Biology, Chinese Academy of Medical Sciences & Peking Union Medical College, Kunming 650118, China; hzl@imbcams.com.cn (Z.H.); yangfenmei@imbcams.com.cn (F.Y.); wenhaiyu@imbcams.com.cn (W.Y.); 3 Key Laboratory of Animal Models and Human Disease Mechanisms, Kunming Institute of Zoology, Chinese Academy of Sciences, Kunming 650223, China; zhoujm@mail.kiz.ac.cn

**Keywords:** herpes simplex virus type 1, rhesus macaques, pathology

## Abstract

As one of the major pathogens for human herpetic diseases, herpes simplex virus type 1 (HSV1) causes herpes labialis, genital herpes and herpetic encephalitis. Our aim here was to investigate the infectious process of HSV1 in rhesus macaques and the pathological features induced during this infection. Clinical symptoms that manifested in the rhesus macaque during HSV1 infection included vesicular lesions and their pathological features. Viral distribution in the nervous tissues and associated pathologic changes indicated the typical systematic pathological processes associated with viral distribution of HSV1. Interestingly, vesicular lesions recurred in oral skin or in mucosa associated with virus shedding in macaques within four to five months post-infection, and viral latency-associated transcript (LAT) mRNA was found in the trigeminal ganglia (TG) on day 365 post-infection. Neutralization testing and enzyme-linked immunospot (ELISpot) detection of specific T cell responses confirmed the specific immunity induced by HSV1 infection. Thus, rhesus macaques could serve as an infectious model for HSV1 due to their typical clinical symptoms and the pathological recurrence associated with viral latency in nervous tissues.

## 1. Introduction

As a major cause of human herpetic diseases, herpes simplex virus type 1 (HSV1) not only results in significant herpes labialis with very high morbidity in populations worldwide, as shown by epidemic studies [1], but it is also an important cause of painful genital herpes and herpetic encephalitis [2,3]. For decades, multiple studies on viral pathogenesis have formed a systematic network of knowledge of HSV1 infection in the host [4,5]. Based on these studies, further investigation into viral pathogenesis in humans requires an animal model to properly mimic whole pathological processes [6]. Previous studies have reported various animal models of HSV1 infection, such as in mice, rabbits and guinea pigs, and have identified differing patterns of acute and latent HSV1 infection [6,7,8,9]. These animal models present nonspecific clinical symptoms and death reflective of viral strain virulence [10] and have provided evidence of pathological changes in the nervous tissues during acute or latent infection [11], while various immune responses have been observed [12].

To date, we have obtained relevant data about acute and latent HSV1 infection in these animals, which contributed to our basic understanding of the viral infectious cycle [13]. However, the limitations of these models, which are not natural hosts of HSV1, have prevented elucidation of viral pathogenesis in humans [14].Recently, a report showed that a new experimental animal, the tree shrew, models HSV1 infection and confirmed latency and reactivation of the virus during infection [15]. However, genomic analyses indicate that all of these animals are genetically distinct from primates, suggesting potential differences in physiology. These findings indicate that additional animal species are needed to better understand the mechanisms of HSV1 latency and reactivation based on the animals’ physiological characteristics.

Because non-human primates share a similar genetic background with humans, they should be considered in detailed investigations of HSV1 viral pathogenesis and for a more restricted animal model in order to study viral treatment and preventive measures. Previously, several reports assessed HSV1 in the owl monkey and rhesus macaques [16,17,18]. Although these works did not continue further because of increasing animal costs and other technical reasons, the genetic similarity of non-human primates to humans suggested that they may be susceptible to a variety of viruses that infect humans and that they may provide valuable data on HSV1 infection. Due to the lack of further studies, several infectious analyses of HSV1 for the non-human primate model were not conducted, including the effective viral dose for an infection, the sensitive age of the animal and even the significant pathological indicators of viral infection. To clarify the clinical features of HSV1 infection in this model and the applicability of this animal for the evaluation of drugs and preventive vaccines of HSV1, we observed HSV1 infection in rhesus macaques and investigated the viral infectious features. The results indicated that rhesus macaques manifest clinical symptoms of vesicular lesions and pathological features similar to those observed in humans during HSV1 infection. Interestingly, the viral distribution in the nervous tissues, the associated pathological changes, the recurring herpetic diseases based on latency, and virus reactivation all suggest a systematic process characterized by HSV1 infection. All observations here indicate the value and significance of rhesus macaques in studies of the infectious mechanism, clinical treatment, and preventive measures for HSV1 infection.

## 2. Materials and Methods

### 2.1. Virus and Cells

The HSV1 F strain was used in this study. The strain was grown in Vero cells (ATCC, Manassas, VA, USA). The virus was harvested and then frozen at −80 °C when typical cytopathic effects (CPE) developed and was produced at a concentration of 10^6.0^–10^7.0^50% cell culture infectious doses (CCID_50_)/mL. The Vero cells were maintained in minimum essential medium (Invitrogen, Carlsbad, CA, USA) containing 10% fetal bovine serum (Gibco, Grand Island, NY, USA) and were used for viral proliferation and titration.

### 2.2. Animal Feeding and Care

The animal experiments were designed based on the principles outlined in the *Guide for the Care and Use of Laboratory Animals* by the National Research Council of the National Academies [19] and the *Guidance to Experimental Animal Welfare and Ethical Treatment* by the Ministry of Science and Technology of the People’s Republic of China (2006) [20]. The experimental protocols were reviewed and approved by the Yunnan Provincial Experimental Animal Management Association (Approval No. SCXK [Dian] 2011-0005) and the Experimental Animal Ethics Committee of the Institute (Approval No. YISHENGLUNZI [2013]4). The animals were reared in cages (Biosafety Laboratory (BSL)-2 conditions) with the room temperature maintained at approximately 25 °C with sufficient fresh air and natural light. Food, water, and fruits were readily available. All animals were fully under veterinarian care at the Institute of Medical Biology (IMB), Chinese Academy of Medicine Science (CAMS). All animals were negative for herpes simian B virus and isolated 2 weeks before starting the study. Prior to animal infection, a neutralization test against HSV1 was performed to confirm that they did not have anti-HSV1 antibodies.

### 2.3. Experimental Design and Sample Collection

Four 1.5-year-old monkeys were treated by scarifying the lip sites with a 24-gauge needle and applying 10^4^ CCID_50_ of HSV1 to each monkey lip. Mock-infected animals were subjected to phosphate-buffered saline (PBS) solution as described above. Various swab samples (from the mouth, nose, eye, feces and urine) of infected monkeys were collected continuously for 10 days after infection and were then centrifuged at 10,000× *g* for 10 min. The supernatants were used for qRT-PCR analysis to determine viral load. Two macaques were anesthetized using ketamine (10 mg/kg of body weight, Phoenix Pharmaceuticals, St Joseph, MO, USA) and were then sacrificed on day 10 (#14065 and #14137) or 365 (#14067 and #14139). Various tissues were homogenized in a Tissue Lyser II system (Qiagen, Hilden, Germany) from the sacrificed animals and were used for qRT-PCR analysis.

### 2.4. Virus Titration

Virus titration was analyzed by performing a micro-titration assay according to a standard protocol [21]. Briefly, viral stocks were serially diluted 10 times and then added to 96-well plates coated with Vero cells. Plates were incubated at 37 °C in 5% CO_2_ and scored for the presence of CPE 7 days post-infection.

### 2.5. Quantitative Detection of Viral Genomic DNA and qRT-PCR Analysis of Viral Transcript mRNA

Viral DNA was extracted from tissues and swabs using an AxyPrep Body Fluid Viral DNA/RNA Miniprep Kit (Axygen, Silicon Valley, USA) according to the manufacturer’s instructions. The qRT-PCR quantification was performed using the pre-mix Ex Taq (probe qPCR) (TaKaRa, Japan) on a 7500 Fast RT-PCR system (Applied Biosystems, Foster City, CA, USA). For detection accuracy, three primer pairs were used for the genes ICP0, UL30, and UL48. Primers and probes for the detection of UL30 were previously reported [22]. The primers for UL48 and ICP0 were as follows:

UL48 (405 bp): forward primer 5′-TACGCCGAGCAGATGATG-3′, reverse primer, 5′-GATGGTAGACCCGTAATTGTT-3′; ICP0 (114 bp): forward primer 5′-GGACCCAGACCACCTTTGGTTG-3′, reverse primer, 5′-GTCAATCAGCACCCACGAGTTC-3′. 

### 2.6. Histopathological and Immunohistopathological Detection

Tissue samples were fixed in 10% formalin, dehydrated through ethanol gradients, and paraffin was then embedded before obtaining 4-µm sections for hematoxylin and eosin (HE) staining and immunohistochemical (IHC) assays. The HSV1 antigen was detected using a rabbit anti-HSV1 polyclonal antibody (Abcam, London, UK), which reacts with all the major glycoproteins present in the viral envelope; the secondary antibody used was a goat anti-rabbit IgG antibody (Abcam, London, UK). Finally, the slides were visualized using light microscopy (Nikon, Tokyo, Japan).

### 2.7. Co-Culture of Nervous Tissues and Vero Cells

Nervous tissues, TG, and pons varolii were removed from the sacrificed monkey 365 days post-infection. Tissues were cut into smaller sections and placed on monolayers of Vero cells. CPE was monitored daily for seven days. If no CPE was detected, cultures were continuously blind passaged for three generations to ensure that no CPE developed. If CPE was observed, cells were harvested, and DNA was extracted from the culture medium for DNA purification and PCR testing for HSV1.

### 2.8. Hybridization of LAT mRNA in Nervous Tissues

RNA probes corresponding to the latency-associated transcript (LAT) region were labeled with digoxigenin-11-UPT (DigU), and detection was performed as previously described [23] using an Enhanced Sensitive ISH Detection Kit I (POD) (Boster, Beijing, China) according to the manufacturer’s instructions. Briefly, tissue samples were fixed in 4% paraformaldehyde in PBS, dehydrated through ethanol gradients, and embedded in paraffin before obtaining 6-µm sections for experiments. After fixation, 30% H_2_O_2_ and methanol were added to the slides to eliminate endogenous peroxidase, after which the slides were treated with pepsin to expose the LAT mRNA. The slides were then treated in pre-hybridization solution for 4 h at 37 °C and subsequently hybridized in hybridization buffer with LAT probes overnight at 37 °C. The slides were washed in different concentrations of saline-sodium citrate (SSC) buffer and were then incubated with anti-digoxigenin antibody conjugated to biotin for 2 h at 37 °C. The slides were developed with 3, 3′-diaminobenzidine (DAB). Finally, the slides were visualized via light microscopy (Nikon, Tokyo, Japan) to detect the presence of LAT mRNA.

### 2.9. ELISpot

Peripheral blood mononuclear cells (PBMCs) were isolated from two monkeys on days 180 and 365 and were treated as previously described [24]. Four peptides ranging from nine to fifteen amino acids long were derived from various genes (gD, gG, gB, gE). The amino acid sequences can be found in the supplementary material (Table S1), which is available on the Immune Epitope Database (IEDB) [25]. Enzyme-Linked ImmunoSpot (ELISpot) was performed for the detection of interferon (IFN)-γ according to the manufacturer’s instructions (Mabtech, Nacka, Sweden).

### 2.10. Statistical Analysis

**Figure 1:**
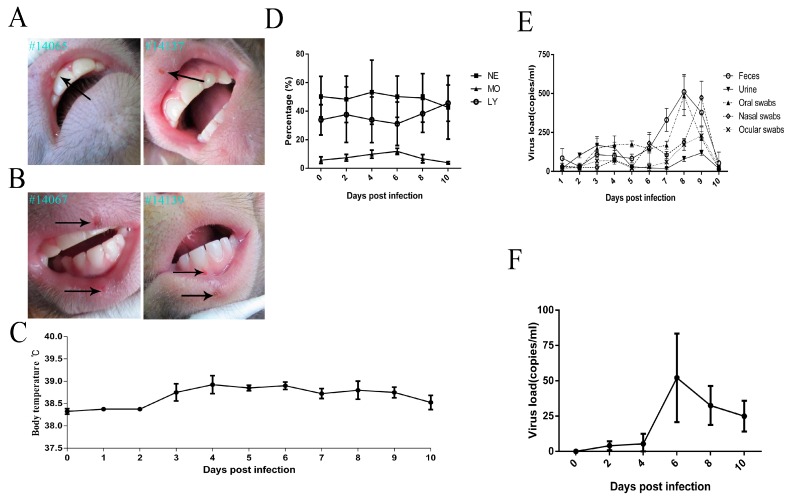
Clinical observation of herpes simplex virus type 1 (HSV1)-infected rhesus macaques. (**A**) vesicle in oral mucosa of macaques, indicated by black arrows; (**B**) multi-vesicles in oral mucosa of macaques, indicated by black arrows; (**C**) body temperatures of infected monkeys were measured by the anal route twice each day post-infection; (**D**) hematological analyses of lymphocytes (LY), monocytes (MO), and neutrophils (NE) from infected macaques; (**E**) feces, urine, oral swabs, nasal swabs and ocular swabs were collected continuously for 10 days after infection and detection of virus shedding. Oral swabs, nasal swabs, ocular swabs and feces indicated the total volume of the eluate; and (**F**) blood samples were collected and assessed for viral load.

**Figure 2:**
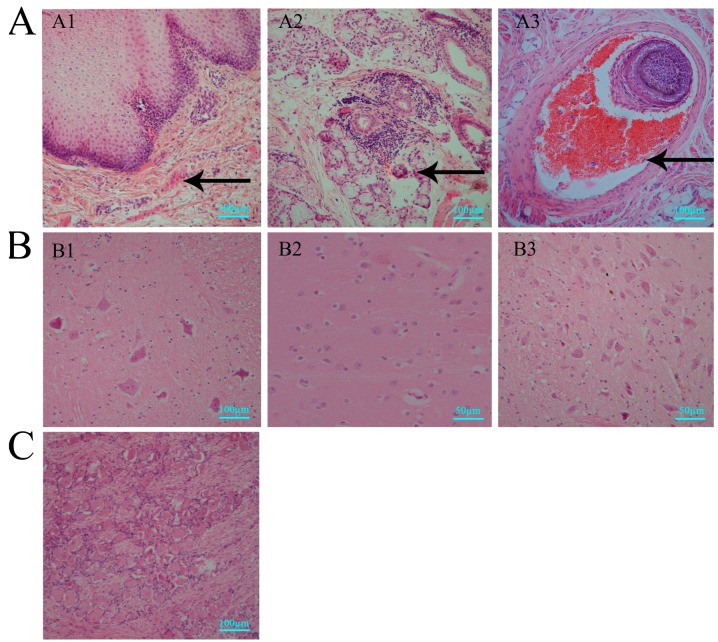
Pathological features of macaques in acute HSV1 infection. (**A**) vesicular lesions in oral skin of the epidermis (A1) (#14065), sebaceous glands (A2) (#14065) and hair follicle (A3) (#14137), structural damage and obvious inflammatory cell infiltration underneath in A1 and A2, and hyperemia in A3; (**B**) nervous tissues, including spinal cord (B1) (#14065), thalamus (B2) (#14065) and mesencephalon (B3) (#14137); no obvious structural damage was observed; (**C**) pathological analysis of TG (#14065); no pathological changes. Positive pathological changes are indicated by black arrows. Scale bars = 50 µm in B2, and B3, scale bars = 100 µm in A, B1, and C.

**Figure 3:**
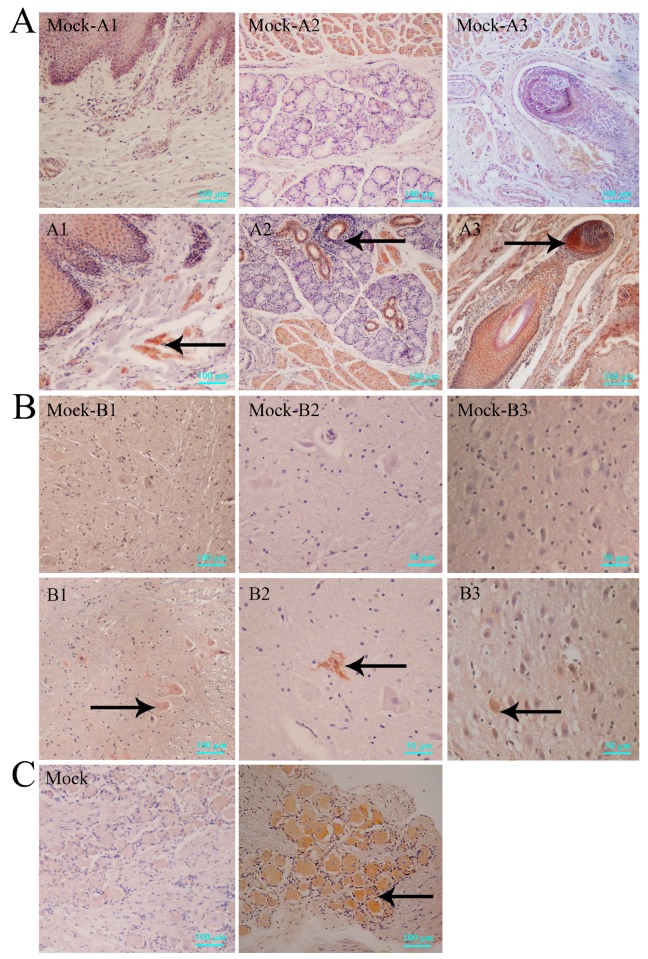
Immunohistochemical (IHC) features of macaques in acute HSV1 infection. (**A**) IHC detection of viral antigens in vesicles of the epidermis (A1) (#14065), sebaceous glands (A2) (#14065) and hair follicle (A3) (#14137); (**B**) IHC detected viral antigens in the spinal cord (B1) (#14065), thalamus (B2) (#14065) and mesencephalon (B3) (#14137); (**C**) IHC analysis of trigeminal ganglia (TG) (#14065); positive viral antigen detection. Positive IHC are indicated by black arrows. Scale bars = 50 µm in B2 and B3, scale bars = 100 µm in A, B1 and C.

Data were obtained from two (Figures 2 and 3) or four macaques (Figure 1C–F) and triplicate experiments (Figures 5C and 7B). The data are expressed as the mean ± SD and were analyzed using Student’s *t*-test. Differences between two groups were evaluated using two-way ANOVA (GraphPad Prism; GraphPad Software, San Diego, CA, USA). Data were considered significant when *p* ≤ 0.05.

## 3. Results

### 3.1. Clinical ManifestationsInduced in Rhesus Macaques Infected by HSV1

Previous reports have indicated that mice may present with redness and swelling of lesions after viral infection at the scratched site [26], and guinea pigs and rabbits easily presented vesicular lesions in genital and ocular sites, respectively [27,28]. However, various viral infectious doses were used in different animals and induced diverse clinical manifestations [13,29]. In our study, the infectious dose used for rhesus macaques was chosen as 10^4^ CCID_50_/animal, which was based on reported data from various animal experiments [23,30,31,32]. Interestingly, all four macaques infected with HSV1 via scratched lip sites presented vesicular lesions within three to five days (Figure 1A). Several individuals showed multi-vesicles in succession that lasted for five to seven days (Figure 1B). Interestingly, this symptom was associated with elevated body temperature in some animals (Figure 1C). Auxiliary hematological analyses suggested no abnormal variations in lymphocytes, neutrophils or monocytes (Figure 1D). Importantly, swabs from the eyes, nasopharynx, anus, and even urine were positive by q-PCR for viral shedding from three to ten days post-infection (Figure 1E). These clinical characterizations are not usually observed in the mouse model [29]. Furthermore, powerful evidence of HSV1 infection in macaques was the viremia that presented within three to ten days (Figure 1F). All of these clinical observations suggested that HSV1 is capable of infecting macaques via scratched oral skin and of inducing clinical manifestations similar to those observed in humans.

### 3.2. Histopathologic Features of Macaques in Acute HSV1 Infection

Pathological studies using mice, rabbits and guinea pigs have suggested that most HSV1 strains are capable of inducing nonspecific inflammatory changes in the epithelium and central nervous system (CNS) tissue [32,33], which can be observed in dead individuals with acute infection [34,35]. These data suggest that histopathological changes due to inflammatory reactions in specific tissues are indicators of acute HSV1 infection in animals.

In our study, histopathological lesions were detected in major tissues, especially in the central nervous system of the infected macaques sacrificed via anesthesia after clinically recording symptoms for ten days post-infection. First, observations of oral tissue sections with vesicular lesions indicated that the vesicles were due to structural damage in the epithelium (Figure 2A1) and sebaceous glands (Figure 2A2), with obvious inflammatory cell infiltration underneath, and were associated with hyperemia in the hair follicles (Figure 2A3). IHC detection using specific antibodies against HSV1 for the same tissue sections showed expression of viral antigens in cells (Figure 3A1–A3). Similar results were observed for the nervous tissues, including the spinal cord (Figure 2B1), thalamus (Figure 2B2) and mesencephalon (Figure 2B3). Pathological analysis of these tissues suggests no obvious structural damage except a focal point of inflammatory cell infiltration, while IHC detected viral antigens (Figure 3B1–B3). Interestingly, similar negative pathological changes (Figure 2C) and positive viral antigen (Figure 3C) detection were identified in the TG, and the viral antigen expression detected in neurons also suggested viral preference to the TG. In contrast, both methods of virus detection in other major organs, including heart, kidney, liver and gallbladder, indicated negative results (Table S2). These data suggested that HSV1 infection in rhesus macaques was capable of inducing vesicular lesions in the oral skin or mucosa and of mediating viral replication at least in nervous tissues through an unknown mechanism without pathological damage.

### 3.3. Virus Distribution in Various Macaque Tissues on Day 10 after HSV1 Infection

**Figure 4:**
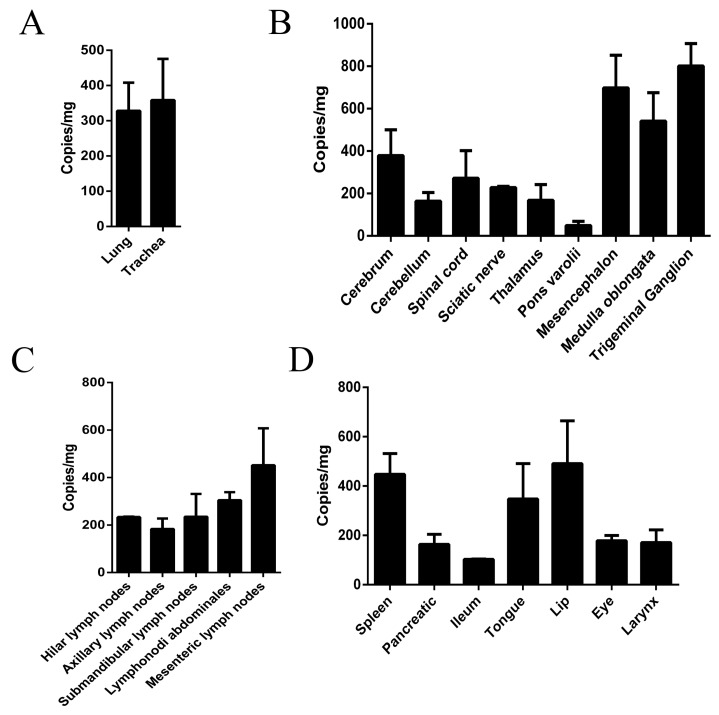
HSV1 distribution in various macaque tissues (No.14065 and No.14137) on day 10 post-infection. HSV1 distribution in tissues include the following: (**A**) respiratory tract, including lung and trachea; (**B**) nervous tissues include cerebrum, cerebellum, spinal cord, sciatic nerve, thalamus, pons varolii, mesencephalon, medulla oblongata and TG; (**C**) lymph nodes include hilar lymph nodes, axillary lymph nodes, submandibular lymph nodes, lymphonodi abdominales and mesenteric lymph nodes; and (**D**) major organs include spleen, pancreatic, ileum, tongue, lip, eye and larynx.

Previous etiological analysis of the HSV1 genome in tissues from infected humans suggested that the virus existed in nervous or non-nervous tissues in a latent state during the infection [36,37]. An identical study in mice also suggested the presence of viral genomes in various tissues post-infection [29]. In our study, we verified viral loads in various tissues from macaques sacrificed via anesthesia on day 10 after infection via q-PCR and virus titration and found high viral loads in the respiratory organs (Figure 4A). However, the high viral distribution in the nervous tissue also included peripheral nerves and the CNS, especially the TG (Figure 4B). While some lymph nodes showed similar viral loads (Figure 4C), various organs presented different viral loads (Figure 4D). These results confirmed previous observations and suggested that HSV1 is capable of replicating in various tissues, especially nervous tissues, in rhesus macaques, similar to that in humans.

### 3.4. Recurrence of Oral Blebs and Associated Virus Shedding in Macaques within Four to Five Months Post-Infection

**Figure 5:**
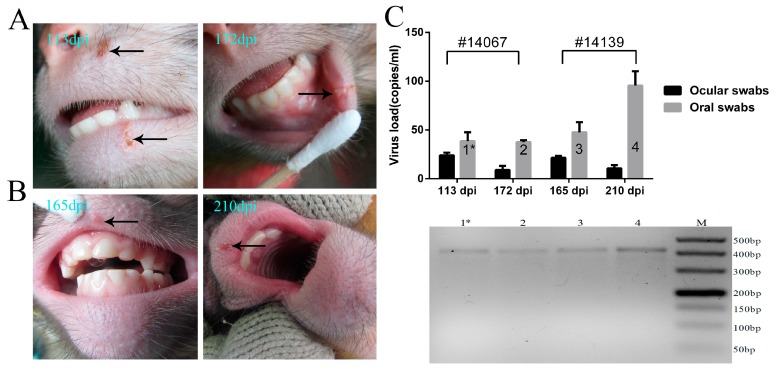
Recurrence of blebs in the oral skin and mucosa within four to five months post-infection. (**A**) spontaneously recurring vesicular lesions on macaque No. 14067 on days 113 and 172; (**B**) spontaneously recurring vesicular lesion on macaque No. 14139 on days 165 and 210; (**C**) oral swabs and ocular swabs were collected from No. 14067 and No. 14139 on recurring vesicular lesion day, and viral shedding was detected; the 1* bar indicates a positive result for viral genomic DNA by PCR in lane 1* (panel below) with primers against UL48 (405 bp); lanes 2, 3 and 4 of PCR electropherograms correspond to 2, 3 and 4 of the graph above, respectively (panel above).

The common pathological features of HSV1 infection in humans include the recurrence of vesicular lesions based on the host physiology [38], which indicates the reactivation of viral replication during its latency in infected neurons followed by epithelial destruction by the reactivated virus [13]. In our experiment, the primary infectious symptoms in the macaques within two weeks post-infection was followed by the restoration to health spontaneously, and the animals remained healthy for four months. Interestingly, further clinical monitoring indicated that vesicular lesions in the oral skin and mucosa of two remaining experimental macaques emerged again on days 113 and 172 and on days 165 and 210, respectively (Figure 5A,B). Swab samples from the mouth and eyes during the corresponding period were positive for viral genomic DNA by PCR using the primers against UL48 (Figure 5C). Further sequencing of the amplified gene fragment obtained by PCR confirmed these results. These data suggest that the recurrence of clinical vesicular lesions associated with viral shedding may be characteristic of the pathological process during HSV1 infection in macaques, although further evidence of the specific relationship between this clinical recurrence and the reactivation of latent viruses in neurons will be needed.

### 3.5. Latent Viral Infection in Macaques on Day 365 Post-Infection

**Figure 6:**
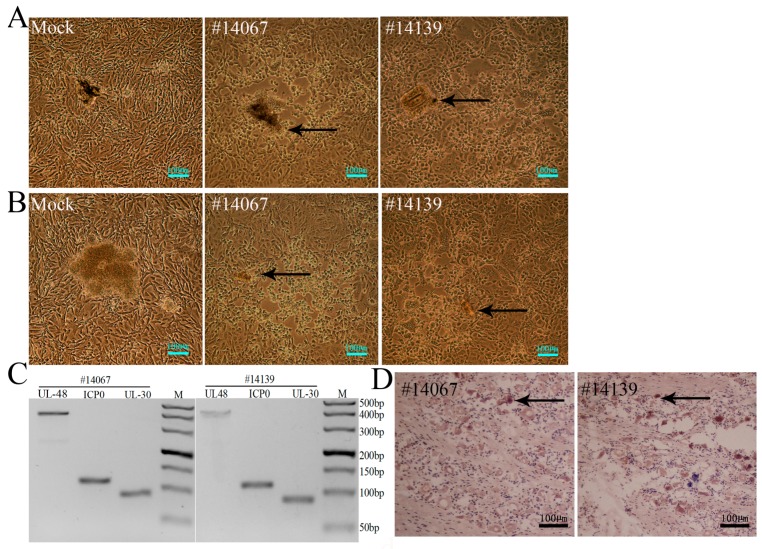
HSV1 was present in nervous tissues in a latent state one year post-infection. (**A**) co-cultured Vero cells with TG of No. 14067 and No. 14139. CPE is indicated by black arrows, scale bars = 100 µm; (**B**) co-cultured Vero cells with pons of No. 14067 and No. 14139. CPE is indicated by black arrows, scale bars = 100 µm; (**C**) viral genome detection of a mixture of TG tissues and co-cultured Vero cells by PCR amplification of DNA with primers against the UL48 (405 bp), ICP0 (114 bp) and UL30 (92 bp) genes; and (**D**) in situ hybridization of TG tissues of No. 14067 and No. 14139 using a specific LAT mRNA probe. Positive in situ hybridization results are indicated by black arrows, scale bars = 100 µm.

The most important event in the pathological process of HSV1 infection in humans is the latent infection of neurons and subsequent reactivation [39]. In this case, our effort to evaluate a macaque model of HSV1 infection focused on whether the viral genome and its transcription were detected in the nervous tissues of infected macaques over a long period of time. Based on the recurrence of herpetic labialis in the two remaining animals within four to five months post-infection, we further investigated all samples collected from nervous tissues, especially TG and other tissues, from these two macaques. These animals were sacrificed via anesthesia on day 365 post-infection, and samples were taken for analysis of their pathological changes and viral transcription. The results indicated no pathological changes in the CNS, peripheral nerves, or other organs (Table S3), as viral genomic DNA was not detected by PCR in these tissue samples (Table S3). However, when these nervous tissues were homogenized and co-cultured with Vero cells, the Vero cells co-cultured with TGs and the pons from the two animals showed typical CPE (Figure 6A,B). PCR analysis for viral genomic DNA with primers against the UL48, ICP0 and UL30 genes indicated the presence of the HSV1 genome in a mixture of TGs and co-cultured Vero cells (Figure 6C). These data indicate the possibility of viral latency in these tissues, but additional studies to determine whether the viral genome could express its functional mRNA are required to support this hypothesis. An additional experiment using a probe against LAT mRNA investigated the expression of LAT mRNA in neurons of TG tissues from the two macaques via in situ hybridization. Assessments of viral LAT mRNA were positive in several neurons of the TG tissues from the two animals (Figure 6D). These results strongly supported the hypothesis of viral latency established in the TG tissues of macaques on day 365 post-infection. Based on these results and the recurrence of clinical vesicular lesions in the oral skin and mucosa within four to five months, we hypothesized that viral latency was established in the TG of infected macaques by HSV1.

### 3.6. HSV1 Infection Is Capable of Inducing a Specific Immune Response in Macaques

**Figure 7:**
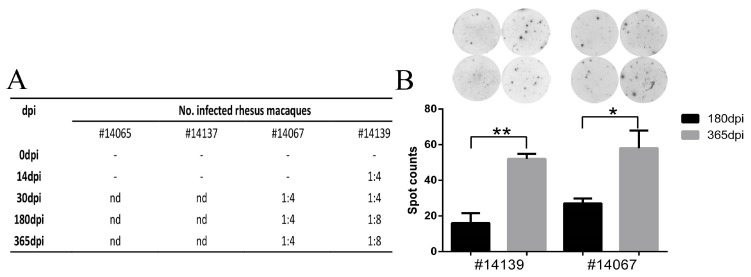
Specific immune responses induced during HSV1 infection in macaques. (**A**) neutralizing antibody testing of serum from macaques No. 14065, No. 14137, No. 14067 and No. 14139. “nd” indicates not detected. No. 14065 and No. 14137 were sacrificed on day 10; and (**B**) ELISpot detection of IFN-γ for specific T cell responses of No. 14067 and No. 14139 on days 180 and 365.“*” indicates *p* < 0.05, “**” indicates *p* < 0.01.

The data collected from HSV1-infected macaques suggested an integrated pathologic process, including acute and latent infectious phases, with clinical features similar to those in humans. These features include viral proliferation in peripheral tissues and viral latency in the nervous tissues, such as the TG. Our results also suggested that a specific immune response against this virus was induced during viral infection based on the interaction of viral components and the host; neutralization tests of serum collected from macaques on days 30, 180 and 365 after viral infection were positive but had low titers (Figure 7A). However, no neutralizing antibody was found in animals No. 14065 and 14137 in serum collected ten days after viral infection (Figure 7A). Interestingly, in an ELISpot analysis for specific T cell responses induced by viral antigen peptide stimulation, greater T cell responses were found in all macaques on days 180 and 365 post-infection (Figure 7B), which suggested that specific immune memory against HSV1 antigens had formed in these animals. Both indicators confirmed the specific immunity induced by HSV1 infection in macaques.

## 4. Discussion

As a viral pathogen with high infectivity for human populations worldwide, HSV1 has long been a concern in different fields [40]. Many studies have described its complicated genomic structure, transcription, and interaction with host cells, suggesting a unique viral pathogenesis mechanism [41,42]. This complexity has made research on clinical treatments and preventive measures for HSV1 infection more challenging than those for other viral pathogens [43] and increased the difficulty of establishing useful animal models of infection [6]. For decades, the mouse, rabbit and guinea pig were used as models for this virus infection and provided much of the data on its viral pathogenesis [44,45,46]. In the last century, reports indicated that the owl monkey and other *Aotus* monkeys were sensitive to HSV1 infection and confirmed viral proliferation in both species [47,48]. Later, Hunter et al. used the New World owl monkey to model and evaluate the immunogenicity and safety of an attenuated HSV1 strain, with positive results [49]. These works demonstrated the significance of non-human primates as appropriate animal models for the study of HSV1 infection and suggested the advantage of non-human primate research in this field based on the fact that non-human primates have genetic similarities to humans. However, other researchers have expressed concerns regarding the ethics of using primates [50]. 

Few analyses have been performed of rhesus macaque models infected by HSV1, and several important parameters for viral infections in this animal remain unknown, such as age susceptibility to infection, infectious pathways, and effective infectious dose [51]. We designed an experiment using rhesus macaques aged eighteen months, equal to six to seven human years of age, that were infected through scratched sites in the oral skin, similar to natural infections in humans, using a viral infectious dose of 10^4^CCID_50_/each. This dose was postulated as a medium dose based on the low dose of 10^3^ pfu used in the New World owl monkey and high dose of 3 × 10^5^ pfu used in mice [13,49]. This experimental design was hypothesized to mimic similar infections in other animals and was evaluated experimentally. Fortunately, the infected macaques developed typical oral blebs, associated body temperature increases, viral shedding in the mouth or eyes, and peak viremia. Generally, human herpetic diseases induced by HSV1 show clinical symptoms, including the typical vesicular lesions in specific sites, especially the skin of the lips or the genital epithelium, which are usually associated with pain [43]. These data and our observations provided evidence supporting the sensitivity of rhesus macaques to HSV1 infection and were consistent with the results of pathological examinations, in which epithelial tissues with vesicular lesions associated with inflammatory cell infiltration in the oral skin or mucosa were found to express viral antigens. The viral replication and slight inflammatory cell infiltration in the nervous tissues observed on day ten post-infection suggested that the virus was transmitted to target tissues, likely through axial retrograde transport along peripheral nerves [37]. In clinical observations of humans infected by HSV1, vesicular lesions in the oral skin and other tissues, where the viral genome can be detected, are associated with histopathological changes [52]. These findings indicated a similarity of the HSV1 infectious process in macaques and humans. Interestingly, the recurrence of vesicular lesions in the oral skin or mucosa associated with virus shedding in macaques within four to five months post-infection and the reactivation of virus from TG samples obtained on day 365 post-infection suggested that viral latency exists in infected macaques. This hypothesis was further confirmed by the positive detection of LAT mRNA transcripts in the TG. Thus, rhesus macaques would be an appropriate species to model HSV1. With its capacity to produce typical clinical symptoms and to exhibit pathologic recurrence based upon viral latency in the nervous tissues, this model could be used for further investigations of viral pathogenesis.

However, due to limited animal resources, we were not able to observe the whole viral infectious process in macaques and lack sufficient data to describe the development of the disease and its outcome. More work is needed to determine the suitable infectious dose, the infectious route of this model, and the relationship of these parameters with pathological processes during HSV1 infection. The results observed here reflect a basic pathological process of viral infection consisting of clinical characterizations in the acute phase of infection and the recurrence of vesicular lesions due to viral latency in the nervous tissues within one year. These observations strongly resemble the pathological features observed in humans. Therefore, more research focused on the pathogenesis of HSV1 infection in macaques will be needed for a comprehensive understanding of this model.

## 5. Conclusions

Rhesus macaques could serve as an infectious model for HSV1 due to their typical clinical symptoms and the pathological recurrence associated with viral latency in nervous tissues. With its capacity to produce typical clinical symptoms and to exhibit pathologic recurrence based upon viral latency in the nervous tissues, this model could be used for further investigations of viral pathogenesis.

## Supplementary Materials

The following are available online at www.mdpi.com/1999-4915/9/2/26/s1. Table S1: Amino acid sequences of peptides; Table S2: Pathological and immunohistochemical analyses of major organs from macaques in the HSV1 acute infectious phase. Table S3: Pathological observation and viral genomic DNA detection in various tissues from macaques on day 365 post-infection.
