# Supplementary material for: The Characteristics of Herpes Simplex Virus Type 1 Infection in Rhesus Macaques and the Associated Pathological Features
Source: Viruses. 2017 Jan 30;9(2):26. doi: 10.3390/v9020026 (PMC5332945; doi:10.3390/v9020026)
Supplement: Supplementary file 1 [file viruses-09-00026-s001.zip › Table S2.docx]

**Table S2** Pathological and immunohistochemical analyses of major organs from macaques in the HSV1 acute infectious phase.

| *No.* | *Pathology and immunohistochemistry* | | | | | |
| --- | --- | --- | --- | --- | --- | --- |
|  | Heart | Kidney | Liver | Gallbladder | Muscles | Esophagus |
| #14065 | - | - | - | - | - | - |
| #14137 | - | - | - | - | - | - |

“-” indicated negative of pathology and immunohistochemistry.
