# Supplementary material for: The Characteristics of Herpes Simplex Virus Type 1 Infection in Rhesus Macaques and the Associated Pathological Features
Source: Viruses. 2017 Jan 30;9(2):26. doi: 10.3390/v9020026 (PMC5332945; doi:10.3390/v9020026)
Supplement: Supplementary file 1 [file viruses-09-00026-s001.zip › Table S3.docx]

**Table S3** Pathological observation and viral genomic DNA detection in various tissues from macaques on day 365 post-infection.

| *No.* | *Pathology and viral genomic DNA detection* | | | | | | | | | |
| --- | --- | --- | --- | --- | --- | --- | --- | --- | --- | --- |
|  | Cerebellum | Ponsvarolii | Mesencephalon | Thalamus | Trigeminal Ganglion | Spinal cord | Nervous dorsalis | Lung | Liver | Spleen |
| #14067 | - | - | - | - | - | - | - | - | - | - |
| #14139 | - | - | - | - | - | - | - | - | - | - |

“-” indicated negative of pathology and viral genomic DNA detection.
