# Supplementary material for: The Characteristics of Herpes Simplex Virus Type 1 Infection in Rhesus Macaques and the Associated Pathological Features
Source: Viruses. 2017 Jan 30;9(2):26. doi: 10.3390/v9020026 (PMC5332945; doi:10.3390/v9020026)
Supplement: Supplementary file 1 [file viruses-09-00026-s001.zip › Table S1 Amino acid sequences of peptides.docx]

**Table S1** Amino acid sequences of peptides.

| *No.* | *Peptide name* | *Sequences* | *No. of amino acids* |
| --- | --- | --- | --- |
| 1 | gD | DASLKMADPNRFRGK | 15 |
| 2 | gG | TSKGRPLVPTPQH | 13 |
| 3 | gB | NLLTTPKFT | 9 |
| 4 | gE | AEMRIYSESLYHPQL | 15 |
